# High Fat Diet Exaggerate Metabolic and Reproductive PCOS Features by Promoting Oxidative Stress: An Improved EV Model in Rats

**DOI:** 10.3390/medicina59061104

**Published:** 2023-06-07

**Authors:** Dejana Rakic, Jovana Joksimovic Jovic, Vladimir Jakovljevic, Vladimir Zivkovic, Maja Nikolic, Jasmina Sretenovic, Marina Nikolic, Nikola Jovic, Marija Bicanin Ilic, Petar Arsenijevic, Aleksandra Dimitrijevic, Tatjana Vulovic, Natasa Ristic, Kristina Bulatovic, Sergej Bolevich, Lazar Stijak, Suzana Pantovic

**Affiliations:** 1 Department of Gynecology and Obstetrics, Faculty of Medical Sciences, University of Kragujevac, 34000 Kragujevac, Serbia; dejavulovic@gmail.com (D.R.); docctorny@gmail.com (N.J.); malaabahelica@gmail.com (M.B.I.); petar.arsenijevic@yahoo.com (P.A.); saskadkg@gmail.com (A.D.); 2 University Clinical Center Kragujevac, Zmaj Jovina 30, 34000 Kragujevac, Serbia; tatjana_vulovic@yahoo.com; 3 Department of Physiology, Faculty of Medical Sciences, University of Kragujevac, 34000 Kragujevac, Serbia; drvladakgbg@yahoo.com (V.J.); vladimirziv@gmail.com (V.Z.); majanikolickg90@gmail.com (M.N.); drj.sretenovic@gmail.com (J.S.); marina.rankovic.95@gmail.com (M.N.); spantovic@medf.kg.ac.rs (S.P.); 4 Center of Excellence for Redox Balance Research in Cardiovascular and Metabolic Disorders, 34000 Kragujevac, Serbia; 5 Department of Human Pathology, I.M. Sechenov First Moscow State Medical University, 119146 Moscow, Russia; bolevich2011@yandex.ru; 6 Department of Pharmacology, I.M. Sechenov First Moscow State Medical University, 119435 Moscow, Russia; 7 Department of Surgery, Faculty of Medical Sciences, University of Kragujevac, 34000 Kragujevac, Serbia; 8 Department of Cytology, Institute for Biological Research “Siniša Stanković”—National Institute of Republic of Serbia, University of Belgrade, 11060 Belgrade, Serbia; negicn@ibiss.bg.ac.rs; 9 Faculty of Medicine, University of Pristina in Kosovska Mitrovica, 38220 Kosovska Mitrovica, Serbia; kristinajakovljevic@gmail.com; 10 Institute of Anatomy, School of Medicine, University of Belgrade, 11000 Belgrade, Serbia; lazar.stijak@gmail.com

**Keywords:** ovary, PCOS, oxidative stress, estradiol-valerate, high-fat diet

## Abstract

*Background and Objectives*: Polycystic ovary syndrome (PCOS) is a frequent multifactorial endocrinopathy affecting women in the reproductive period, often associated with infertility and metabolic disorders. The use of animal models helps to better understand etiopathogenesis, enabling the examination of the effects of certain drugs in order to discover the best possible therapeutic approach. We tried to investigate the additional effect of estradiol-valerate (EV) and high-fat diet (HFD) in female rats to explore PCOS-related alterations with special focus on oxidative stress. *Materials and Methods*: Animals were divided into three groups: control group (CTRL, n = 6), estradiol-valerate group (EV, n = 6), and estradiol-valerate group on HFD (EV + HFD, n = 6). PCOS was induced by single subcutaneous injection of long-acting EV in a dose of 4 mg/per rat. We tried to improve the metabolic characteristics of the PCOS animal model by adding HFD, so the CTRL and EV group had a regular diet, while the EV + HFD group had HFD during the induction period of 60 days. *Results*: We observed alterations of anthropometric parameters and hormonal disturbances, along with estrus cycle impairment reassembly to obese-type PCOS phenotype. Moreover, glucose metabolism was impaired after addition of HFD to EV protocol, contrary to EV administered alone. Histological analysis confirmed more numerous cystic follicles after the combination of EV and HFD protocol. The alterations of oxidative stress markers could be related to and serve as the mechanistic base for development of PCOS-related endocrine, reproductive, and metabolic properties. *Conclusions*: The additive effect of EV and HFD was obvious in the majority of the parameters observed. Our study strongly demonstrated metabolic as well as reproductive properties of PCOS in rats.

## 1. Introduction

Polycystic ovary syndrome (PCOS) is a frequent multifactorial endocrinopathy affecting women in the reproductive period, often associated with infertility and metabolic disorders. The incidence of PCOS is between 5 and 20% of the population, depending on the diagnostic criteria [1]. Since 1935, when Stein and Leventhal presented a group of seven women with common complaints—disorder of the menstrual cycle, hirsutism, and enlarged ovaries with the presence of numerous small follicles—PCOS underwent changes in definition and classification. Today, PCOS is described as a phenomenon of the 20th century, as a silent epidemic is happening nowadays [2,3]. In the last decades, numerous studies have been conducted regarding PCOS, although there are still many ambiguities and contradictions regarding etiopathogenesis, therapy, and its relation to cardiovascular, psychiatric, esthetic, and other features [4].

The etiopathogenesis of PCOS is very complex and despite numerous theories such as low-grade inflammation and oxidative stress (OS), it remains unclear; it is believed to be based on the synergism of polygenetic, epigenetic, and environmental factors [5]. According to the Rotterdam criteria, PCOS represents the presence of two out of three criteria: hyperandrogenism (clinical or biochemical), oligo or anovulation, and ultrasonographically verified polycystic ovarian morphology. PCOS phenotypes according to the Rotterdam criteria are as follows: type 1: hyperandrogenism or hirsutism, and ovarian dysfunction (irregular, anovulatory menstrual cycles) and polycystic ovaries; type 2: hyperandrogenism and hirsutism, and irregular anovulatory menstrual cycles; type 3: hyperandrogenism, hirsutism, and presence of ovarian cysts; and type 4: irregular anovulatory menstrual cycles and the presence of ovarian cysts. Hyperandrogenism is an entity that occurs in 80% of PCOS cases. Interestingly, in obese women, the prevalence of PCOS is higher and amounts to between 38 and 88% [6]. Insulin resistance and diabetes mellitus type 2 (DMT2) are often associated with PCOS, increasing the risk of cardiovascular diseases, dyslipidemia, abdominal obesity, and endometrial cancer. Hyperinsulinemia increases the number of receptors for luteinizing hormones (LH) on the granulosa cells of the ovaries and they produce androgens, the production of globulin that binds sex hormones (SHBG) in the liver is reduced, and androgens are elevated and free in the circulation. Low values of SHBG are associated with impaired glucose metabolism and the consequent DMT2 development. Under the influence of insulin, the ratio of follicle-stimulating hormone (FSH) and LH changes and the level of LH increases significantly. Inhibition of FSH and insulin-like growth factor I (IGF1) stimulates steroidogenesis in granulosa cells. The dominant follicle does not develop, but all follicles remain at the preantral and antral stage, which lead to an increase in the volume of the ovaries (>10 mL), while LH hypersecretion stimulates the cells to produce androgens and reduces the effect of FSH. As a result, premature luteinization occurs by increasing gonadotropin-stimulated estradiol and progesterone preventing the maturation and growth of follicles [7].

By analyzing the level of pro-oxidant molecules in blood and tissues, such as malondialdehyde, nitric oxide, xanthine oxidase, hydrogen peroxide, superoxide anion radical, as well as the activity of antioxidant enzymes such as superoxide dismutase (superoxide dismutase—SOD) and catalase (catalase—CAT), glutathione reductase, and glutathione peroxidase, we come to the conclusion that the level of oxidative stress in PCOS patients increases [8]. In addition, studies on animals in which PCOS is induced by different methods, show that the level of prooxidants in PCOS is significantly higher compared to control values, and that the antioxidant capacity is reduced [9]. Various antioxidant protocols were used to improve reproductive functions in male and female rats [10,11,12], confirming the role of OS in their pathophysiological alterations. OS can be conditioned by genetic and environmental factors. In general, in the field of OS, PCOS is associated with cell apoptosis triggered by mitochondrial damage, but also with point mutations of genes that are associated with metabolic complications [13]. Furthermore, OS can act on various signaling pathways that are important for the homeostasis of the reproductive system.

The importance of PCOS and its complex and unclear etiopathogenesis are the reason for the existence of numerous preclinical and clinical investigations; despite this, there is a lack of adequate therapy. To date, numerous experimental models of PCOS have been developed, using different animals such as rodents, sheep, and rhesus monkeys [14]. Rodents are most often used for practical reasons, although there are certain limitations in the development of complete clinical manifestations of PCOS. Rodent models of PCOS have provided particularly valuable insights into the pathogenesis of PCOS due to their ease and accessibility of use, short reproductive and lifespan, and high genetic similarity to the human genome as well as the feasibility of generating genetically adapted mice. The use of animal models helps to better understand etiopathogenesis (although it is not the same as in humans), enabling the examination of the effects of certain drugs in order to determine the best possible therapeutic approach. The PCOS model in rats can be induced by hormone or enzyme inhibition, prolonged light exposure, high-calorie diet, and transgenic technology. In addition, PCOS can be induced by the administration of androgens—dehydroepiandrosterone (DHEA), testosterone propionate (TP), 5a-dihydrotestosterone (DHT) as well as long-acting estradiol-valerate (EV), antiprogesterone–mifepristone, and aromatase inhibitor–letrozole in female rats [15].

PCOS can be induced by a single application of EV, causing anovulation, and the morphology of polycystic ovaries after 60 days, which is more comfortable and less stressful for the animals than daily manipulation and the application of hormones, as is necessary during the application of androgens [16,17]. Application of a supraphysiological dose of long-acting estrogen disturbs LH storage and secretion, causing impairment of the pituitary-ovarian axis and producing the PCOS-like properties in rats [18]. Zangeneh et al. believe that the changes after the application of a single dose of EV occur due to the effect on the peripheral sympathetic neurons that innervate the ovary, lowering the sympathetic activity [19].

On the other hand, a high-calorie, high-fat diet (HFD) causes an inflammatory response leading to abnormal glucose and lipid metabolism. It has been proven that HFD causes metabolic changes such as insulin resistance, hyperinsulinemia, dyslipidemia, obesity, increased levels of progesterone (P), testosterone (T), and LH, irregular menstrual cycles, and polycystic ovarian morphology in rats [20]. HFD causes changes in the ovaries, increasing the number of cystic follicles and thinning the follicle wall, reducing reproductive capacity. The disorder exists in steroidogenesis and folliculogenesis and changes the expression of ovarian tissue genes. The development of changes is slow but progressive and approaches the clinical form of PCOS [21]. Researchers try to find the best animal model, which will encompass more reproductive and metabolic features, and help to simulate pathophysiological milieu as in PCOS patients. To our knowledge, this is the first study investigating the additional effect of EV and HFD in female rats to explore PCOS-related alterations with a special focus on OS.

## 2. Materials and Methods

### 2.1. Ethics Committee Approval

The research was carried out at the Faculty of Medical Sciences of the University of Kragujevac with the approval of the ethics committee (decision number 01-10636, date 4 October 2022). All procedures were conducted according to the guidelines of the European Council directive and according to good laboratory practice and the ARRIVE guide.

### 2.2. Animals

Adult Wistar albino female rats (n = 18, body weight (BW) 150–170 g) were obtained from the Military Medical Academy (Belgrade, Serbia). All animals were placed in cages (3 animals per cage), with free access to water and food, at a temperature of 23 °C and under a cycle of 12 h dark, 12 h light, with the light switched on at 8:00 a.m. After the adaptation period, it was checked whether the animals were in an estrous cycle and only those that had two regular consecutive estrous cycles, lasting for 4–5 days, were subjected to the experimental procedure.

### 2.3. Induction of PCOS

All regularly cycling animals were divided into 3 groups: control group (CTRL, n = 6), EV group (EV, n = 6), and estradiol-valerate group on HFD (EV + HFD, n = 6). PCOS was induced by single subcutaneous injection of long-acting estradiol-valerate in a dose of 4 mg/per rat in 0.2 mL of oil solution [22]. In order to develop polycystic ovary syndrome, it is necessary to wait 60 days after one injection of long-acting estradiol-valerate [23]. We tried to improve the metabolic characteristics of the PCOS animal model by adding HFD, so the CTRL and EV group had a regular diet, while the EV + HFD group had HFD during the induction period of 60 days. The high-calorie diet was 5.24 kcal/g, with 60% of calories from fat [24]. Animals of the control group received vehicle during the experimental protocol (0.2 mL of the oil solution). During the experiment, the animals’ body mass was measured weekly. After the 60 days required for induction, all animals were anaesthetized and sacrificed, while blood and ovarian tissue were collected for further analysis. The euthanization was performed in the same phase of estrus cycle for all animals (estrus phase). Two days before sacrificing the animals, an oral glucose tolerance test (OGTT) was performed, and the day after, an ultrasound examination of the ovaries was performed.

### 2.4. Estrus Cycle Analysis

Determination of the estrous cycle phase was established based on vaginal swabs for last 12 consecutive days of the experimental protocol. In the morning between 9:00 and 10:00 a.m., vaginal lavage was performed with a pipette and the secretion sample was placed on a glass slide soaked in hematoxylin and then analyzed under a light microscope. The estrous cycle phases were confirmed based on the predominance of specific cells. Characteristics were as follows: proestrus—round, nucleated cells; estrus—squamous cells; metaestrus—squamous cells and leukocytes; and diestrus—more nuclear epithelial cells and leukocytes [25].

### 2.5. Oral Glucose Tolerance Test (OGTT)

At 48 h before sacrifice, all animals were subjected to fasting for 12 h and then they were given a glucose gavage in a dose of 2 g/kg. Blood samples were taken at 30, 60, and 120 min after glucose administration. Glucose level was measured with a glucometer (Accu-Chek, Roche 165 Diagnostics, Indianapolis, IN, USA) with appropriate strips.

### 2.6. Ultrasound Examination of the Ovaries

Before the sacrificing, an ultrasound examination was performed on a Hewlett Packard Sonos 5500 device (Andover, MA, USA) with a frequency of 15.0 MHz and a linear transducer. First, all rats were anaesthetized using ketamine (50 mg/kg) and hydralazine (10 mg/kg). Using two-dimensional transabdominal ultrasound, the ovaries were visualized behind the kidneys. Ovaries were measured in three dimensions to determine their volume, as well as longitudinal (D1), transverse (D2), and antero- posterior (D3) diameters. Ovarian volume was calculated by the following formula [volume = π/6 (LOD × TOD × APOD)] [26].

### 2.7. Animal Euthanization and Sample Collection

After the execution of the protocol, all animals in the estrous cycle (the estrous cycle was selected in order to avoid the influence of the cycle on monitoring the parameters) were anesthetized intraperitoneally with the application of a combination of ketamine in a dose of 100 mg/kg and xylazine in a dose of 10 mg/kg. The animals were euthanized by decapitation on guillotine, and trunk blood was collected in the two separate tubes. Following blood coagulation in tubes free of anticoagulants for 2 h at room temperature, serum was obtained by centrifugation at 3500× *g* for 15 min at 4 °C. The clear supernatant was maintained at −20 °C until analysis. Plasma was obtained after centrifuging blood in tubes containing sodium citrate. After removing plasma from the tubes, erythrocyte lysate was obtained by lysis of erythrocyte suspension by 3 volumes of ice-cold distilled water. The samples of plasma and erythrocyte lysate were stored at −20 °C for future analysis [12,27]. The ovaries were isolated for further histological analysis.

### 2.8. Biochemical Analyses

Serum samples were used to determine the levels of testosterone, progesterone, and estradiol. The levels of testosterone, estradiol, and progesterone were determined with an Elecsys 2010 analyzer using the electrochemical immunoassay (ECLIA) method. Standard commercial kits (Elecsys Testosterone II, Progesterone II, and Estradiol III Roche Diagnostics, Mannheim, Germany) were used. Testosterone and progesterone were expressed in ng/mL, while estradiol was expressed in pg/mL. The sensitivities for testosterone, progesterone, and estradiol were 0.025 ng/mL, 0.03 ng/mL, and 5 pg/mL, respectively. The inter- and intra-assay coefficients of variation for testosterone, progesterone, and estradiol were 3.8%, 3%, and 2.2%, 5%, and 3.9%.

### 2.9. Oxidative Stress (OS) Parameters

The following parameters were determined in the plasma of the samples: lipid peroxidase index (expressed as thiobarbiturate reactive acid substance-TBARS), nitrates (NO_2_^−^), hydrogen peroxide (H_2_O_2_), and superoxide anion radical (O_2_^−^) levels. Enzyme activity (superoxide dismutase (SOD) and catalase (CAT)), as well as the non-enzymatic (GSH) antioxidant system, were registered from the lysed erythrocytes.

#### 2.9.1. TBARS Determination

The procedure was carried out by mixing 0.8 mL of the sample and 0.4 mL of trichloroacetic acid. After 15 min of being on ice and centrifugation at 6000× *g* rpm, the supernatants were stored. Next, 1% thiobarbituric acid in 0.05 NaOH was separated with the supernatant at 100 °C for 15 min. The measurement was carried out at 530 nm wavelength. A distilled water solution was used as a blank [28].

#### 2.9.2. Determination of NO_2_

The NO_2_- level was regulated using the NO production index with the Griess reagent [29]. We preserved 0.1 mL of 3 N perchloric acid, 0.4 mL of 20 mM ethylenediaminetetraacetic acid, and 0.2 mL of the sample on ice for 15 min and then centrifuged for 16 min at 6000× *g* rpm. After pouring off the supernatant, 220 μL of K_2_CO_3_ was added. NO_2_ was measured at a wavelength of 550 nm. The distilled water solution was used as a blank.

#### 2.9.3. Determination of H_2_O_2_

The determination of hydrogen peroxide (H_2_O_2_) is formed on the oxidation of phenol with the help of hydrogen peroxide, in a reaction catalyzed by peroxidase (HRPO). A sample of 200 μL of plasma was mixed with 800 μL of freshly prepared red fresh phenol solution and then 10 μL (1:20) of HRPO was added. The H_2_O_2_ level was measured at 610 mm light length and distilled water was used as a blank [30].

#### 2.9.4. Determination of O_2_

Determination of super oxide radicals (O_2_) was measured after reaction with nitro blue tetrazolium in TRIS buffer with the plasma samples. The determination was carried out at a wavelength of 530 mm and distilled water was used as a blank test [31].

#### 2.9.5. Determination of CAT Activity

For the CAT measurement, 50 L of CAT buffer, 100 L of the sample, and 1 mL of 10 mM H_2_O_2_ were combined. The detection was done at a 360 nm wavelength. The quantity of CAT was expressed as U/g of hemoglobin 10^3^ [32], while distilled water served as a blank probe.

#### 2.9.6. Determination of SOD Activity

Misra and Fridovich [33] used the epinephrine technique to determine SOD activity. We added 100 L of epinephrine in the tube after mixing of 100 L of sample with 1 mL of carbonate buffer. The activity of SOD was measured at a wavelength of 470 nm, and it was expressed as U/g of hemoglobin 10^3^.

#### 2.9.7. Determination of GSH Concentration

Based on GSH oxidation by 5,5-dithiobis-6,2-nitrobenzoic acid, the quantity of reduced glutathione (GSH) was measured. The preparation of the GSH extract involved mixing 0.1 mL of 0.1% EDTA, 400 mL of plasma, and 750 mL of precipitation solution (1.67 g of metaphosphoric acid, 0.2 g of EDTA, 30 g of NaCl, and 100 mL of distilled water). After 15 min of extraction on ice and mixing in a vortex device, the mixture was centrifuged at 4000× *g* rpm for 10 min. Distilled water was used as a blank probe and measurements were made at a wavelength of 420 nm [34].

### 2.10. Histological Analysis of Ovary

After sacrificing the rats, the left ovaries were isolated, cleaned of surrounding tissue, measured, and fixed in 10% formalin, and then analyzed under a light microscope, dehydrated in high alcohol, cleared with xylene, and embedded in paraffin. Dissected tissue sections with a thickness of 4 μm were stained with hematoxin and eosin. Three sections of the ovaries were used to monitor the evaluation of ovarian histomorphology, the number of follicular cysts, and the number of corpora lutea. Analysis was performed using an Olympus BX-51, Olympus Europa GmbH, Hamburg, Germany [26].

### 2.11. Statistical Analysis

Values are introduced as mean ± standard error. The study by Barzegar and colleagues was referenced to calculate the sample size for estradiol-valerate-induced PCOS; the difference between control and PCOS group in number of antral follicles was used for determination of sample size [35]. Each study group was determined to include at least four rats to achieve a power of 90% with 5% alpha error. We increased the number of experimental animals to ensure their necessary number in the case of possible loss of some animals during the experimental protocol. Sample size and power calculations were performed using G*Power 3.1.9.4.

Before statistical processing, all data were subjected to the test of normality depending on the distribution. The data were monitored using the ANOVA (with LDS post-hoc analysis) when distribution was normal, or Kruskal–Wallis test (with Mann–Whitney post-hoc analysis) when distribution was not normal. The estrus cycle was presented by percentages, analyzed using the Chi-square test, and differences were evaluated by the *z*-test. These analyses were conducted using the SPSS statistics program. Values of *p* below 0.05 were considered a statistically significant difference, while values below 0.01 were considered a very significant statistical difference.

## 3. Results

**Figure 1:**
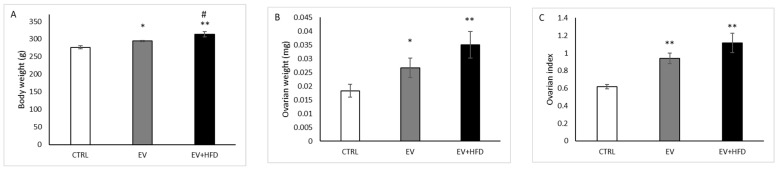
BW (**A**), ovarian weight (**B**) and ovarian index (**C**). Bars represent means ± SEM. * statistical significance at level *p* < 0.05, compared to CTRL; ** statistical significance at level *p* < 0.01, compared to CTRL; # statistical significance at level *p* < 0.05, compared to EV.

As shown in Figure 1A, BW increased in the EV group (*p* < 0.05) and in the EV + HFD group (*p* < 0.01) compared with CTRL. However, there was a significant increase in BW in the EV + HFD group compared with EV (*p* < 0.05). Ovarian weight (Figure 1B) and ovarian index (Figure 1C) were significantly higher (*p* < 0.01) in the EV as well as in EV + HFD groups, when compared with CTRL, while there were no differences between the EV and EV + HFD groups.

**Figure 2:**
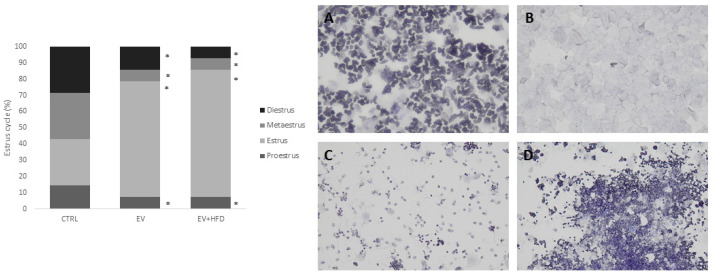
Left: the proportion of different estrous cycle stages in the three groups was analyzed using the Chi-square test, and differences were evaluated by *z*-test. * *p* < 0.05 vs. CTRL. Right: photographs showing different phases of estrus cycle in rats ((**A**)—proestrus; (**B**)—estrus; (**C**)—metaestrus; (**D**)—diestrus).

Rats from the CTRL group had a regular estrus cycle consisting of 4–5 days, while rats from the EV and EV + HFD groups did not shift phases regularly and expressed cessation of cycle in the last 12 days of the conducted protocol. The cycle was arrested dominantly in an estrus phase which was demonstrated by persistent vaginal cornification (PVC) at vaginal smears analysis (Figure 2).

**Figure 3:**
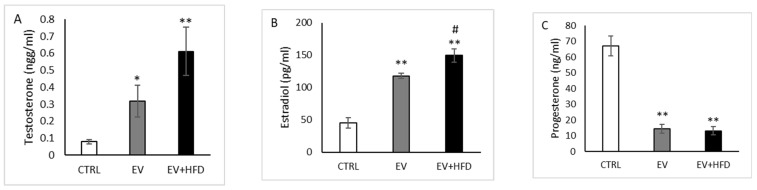
Serum levels of sex hormones. (**A**)—Levels of testosterone; (**B**)—levels of estradiol; (**C**)—levels of progesterone. Bars represents means ± SEM. * statistical significance at level *p* < 0.05, compared to CTRL; ** statistical significance at level *p* < 0.01, compared to CTRL; # statistical significance at level *p* < 0.05, compared to EV.

Serum level of testosterone was significantly higher in the EV group (*p* < 0.05) and in the EV + HFD group (*p* < 0.01) when compared with CTRL (Figure 3A). Serum level of estradiol was significantly higher in the EV group and in the EV + HFD group (*p* < 0.01) when compared with CTRL. Moreover, the EV + HFD group expressed higher level of estradiol compared with the EV group (*p* < 0.05), as shown in Figure 3B. Progesterone level significantly decreased in both EV and EV + HFD groups (*p* < 0.01), as shown in Figure 3C.

**Figure 4:**
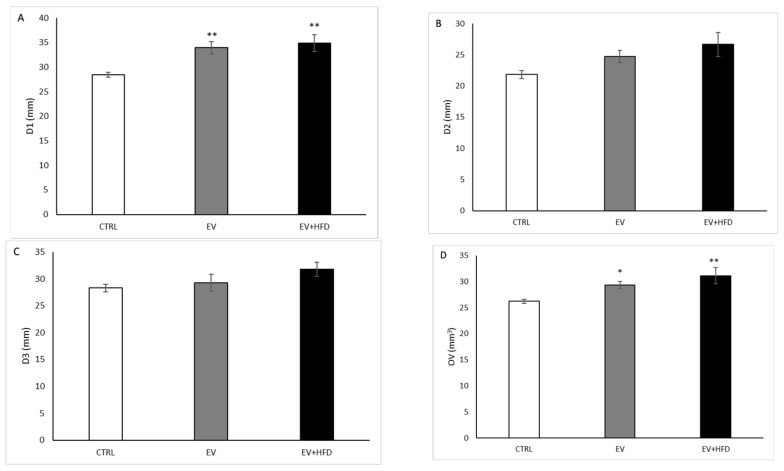
Ultrasound examination of rat’s ovary. (**A**)—longitudinal diameter of the ovary; (**B**)—transversal diameter of the ovary; (**C**)—antero-posterior diameter of the ovary; (**D**)—ovarian volume. Bars represents means ± SEM. * statistical significance at level *p* < 0.05, compared to CTRL; ** statistical significance at level *p* < 0.01, compared to CTRL.

As demonstrated in Figure 4, D1 was significantly higher in both PCOS groups, EV and EV + HFD (*p* < 0.01), compared with the non PCOS–CTRL group. On the other hand, D2 and D3 diameters of ovary were not altered in EV and in EV + HFD when compared with the CTRL group. However, OV was significantly higher in the EV group (*p* < 0,05) and also in the EV + HFD group (*p* < 0.01) compared with control values. Nevertheless, a difference between EV and EV + HFD was not registered in the mentioned ultrasound parameters.

The EV protocol alone did not influence blood glucose levels in basal conditions (fasting blood glucose levels). Moreover, during OGTT, glucose levels did not differ between the EV and CTRL group. We registered higher blood glucose levels after 30 min of OGTT in the EV + HFD group compared with control values. The EV + HFD group showed a higher level of blood glucose compared with the EV group after 30 and after 60 min of OGTT. After 120 min, blood glucose levels were similar in all investigated groups.

As shown in Table 1, index of lipid peroxidation and level of O_2_^−^ were expressed in the highest level in the EV + HFD group (*p* < 0.01 compared with CTRL; *p* < 0.05 compared with EV). Level of nitrites were significantly higher in the EV group compared with CTRL (*p* < 0.05), while a more pronounced increase was observed in the EV + HFD group compared with CTRL (*p* < 0.01). However, the HFD protocol was responsible for a significant increase in the level of nitrites in the EV + HFD group compared with the group where EV was administered alone (*p* < 0.01). H_2_O_2_ levels were significantly lowest in the EV + HFD group (*p* < 0.01 compared with CTRL; *p* < 0.05 compared with EV). Regarding antioxidant capacity, our results showed a gradual decrease in SOD activity with significantly lowest values in the EV + HFD group (*p* < 0.05 compared with CTRL; *p* < 0.05 compared with EV). The activity of CAT was lower in the EV group compared with CTRL (*p* < 0.01) and in the EV + HFD group compared with CTRL (*p* < 0.05). On the other hand, levels of GSH were increased in the EV group compared with CTRL (*p* < 0.05), while the EV + HFD group expressed levels of GSH similar to the CTRL group. However, levels of GSH were significantly lower compared with the EV group (*p* < 0.05).

**Figure 5:**
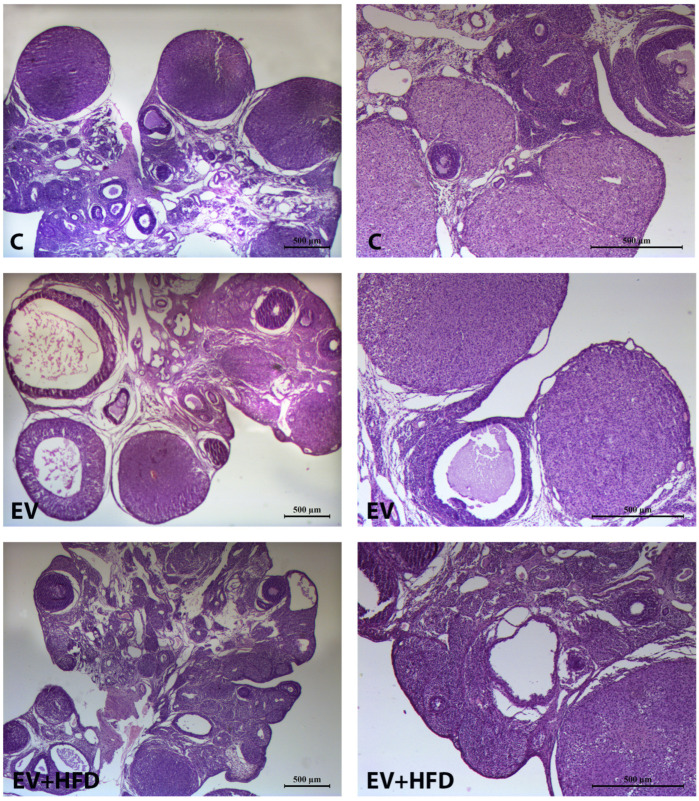
Photomicrographs of ovarian central sections. C—control group; EV—PCOS group, treated by estradiol-valerate; EV + HFD—PCOS group treated by estradiol-valerate and high fat diet. Left column—magnification 2.5×; Right column—magnification 5×.

As presented in Figure 5, ovaries from the C group appeared as healthy structures, with various stages of follicular development (primodrial, primary, secondary, tertiary follicles). No cystic formations were observed in the C group, while corpora lutea were present as markers of previous ovulations. On the other hand, PCOS groups (EV and EV + HFD) presented with remarkably higher number of cystic follicles, enlarged stroma, and lower number of corpora lutea compared with the C group. Moreover, visible atretic follicles were present in higher amount in the EV and EV + HFD groups, while the C group showed physiological process of atresia in ovaries.

**Figure 6:**
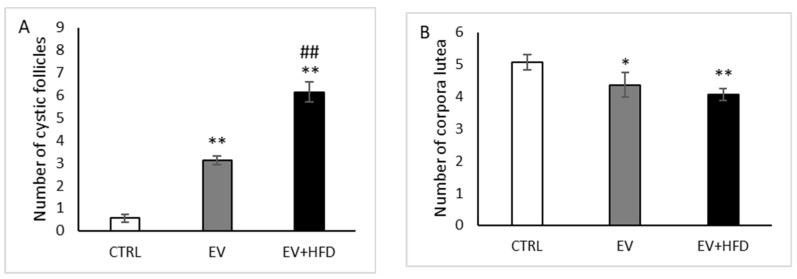
Number of cystic follicles and corpora lutea in rats. (**A**)—number of the cystic follicles; (**B**)—numbers of the corpora lutea. Bars represents means ± SEM. * statistical significance at level *p* < 0.05, compared to CTRL; ** statistical significance at level *p* < 0.01, compared to CTRL; ^##^ statistical significance at level *p* < 0.01, compared to EV.

The number of cystic follicles increased significantly after both type of protocols (*p* < 0.01) compared with the CTRL group. However, the EV + HFD group expressed greater number of cysts compared with the EV group (*p* < 0.01), as shown in Figure 6A. Regarding the number of corpora lutea, EV administered alone led to significant reduction of these formations (*p* < 0.05), as well as EV along with HFD protocol (*p* < 0.01). However, there was no observed difference between the EV and EV + HFD group in the number of corpora lutea (Figure 6B).

## 4. Discussion

The present study investigated the role of HFD along with EV in the development of PCOS-related alterations in a rat experimental model. Our study strongly demonstrated metabolic as well as reproductive properties of PCOS in rats. We observed anthropometric parameter alterations and hormonal disturbances, along with estrus cycle impairment reassembly to obese-type PCOS phenotype. Moreover, glucose metabolism was impaired after addition of HFD to EV protocol, contrary to EV administered alone. Histological analysis confirmed the more numerous cystic follicles after combination of EV and HFD protocol. The alterations of OS markers could be related to and serve as the mechanistic base for development of PCOS-related endocrine, reproductive, and metabolic properties. The additive effect of EV and HFD was obvious in the majority of the parameters observed.

There are numerous protocols for PCOS induction in rodents in the literature; from the prenatal androgenization, by administration of pre- and post-pubertal hormones, to environmental and diet manipulations [36]. Although all mentioned experimental protocols have some advantages compared to human disease (controlled conditions, ability for repetition, harvesting tissue for further analysis), they are lacking in pathophysiological equivalency and PCOS animal models mimic only symptoms and certain aspects of this complex disorder. The mechanism by which EV induces PCOS-like features in animal models was previously described in detail [37]. A single high dose of EV induced a reliable PCOS model in rats, confirmed in many studies [38]. On the contrary, there are studies supported the opposite standing, where EV administration in rats does not represent an adequate animal model to represent PCOS-related changes in humans, as well as reliable pathophysiological settings favorable to explore different therapeutic regimens [39]. However, the present results confirmed mild PCOS-related changes, which were exaggerated by the addition of HFD during 60 days of the protocol. Previous investigation also confirmed that letrozole combined with the HFD protocol augmented alterations of endocrine, metabolic, and reproductive parameters in PCOS rat model [40]. As in our study, adding HFD to the well-established protocol for PCOS induction led to improvement of pathophysiological settings mimicking PCOS in humans. However, our protocol had an advantage because a single dose of intramuscular injection of EV brings less stress to an animal compared with daily administration of letrozole by oral route of administration, decreasing stressful handling of animals, which is known to influence hormone alterations and possible consecutive estrus cycle irregularities. A single injection of EV is a widely used protocol to produce PCOS manifestations in rats, causing polycystic ovary morphology, irregular estrous cycles, changes in basal and pulsatile LH and FSH concentrations, and an increased androgen response to stimulation by human chorionic gonadotropin [41]. On the other hand, the HFD protocol could have led to PCOS-related alterations in female rats [20], as well as a high-fat, high-sugar (HFHS) diet [42], resulting in both metabolic and reproductive characteristics of PCOS in pre-pubertal rats. However, researchers revealed that HFD protocols in post-pubertal age also aggravate ovarian function, particularly in combination with letrozole or DHEA [40,43]. For the first time, our study demonstrated the use of a combination of EV and HFD to produce PCOS-like manifestations in rats, accentuating reproductive and metabolic aspects of PCOS, while minimizing the stressful handling of the animals.

Obesity is believed to play a central role in the development of PCOS, as many women with the condition are reported to be overweight or obese. There is a strong correlation between PCOS and obesity. Factors such as insulin resistance, hyperandrogenemia, and body fat distribution were investigated in obese and non-obese PCOS patients. Most studies could not determine whether PCOS contributed to obesity or vice versa. Obesity may be an important predictor of PCOS. In women predisposed to PCOS, the metabolic and hormonal problems that are present, such as insulin resistance and hyperandrogenism, can lead to weight gain and eventually obesity. Obesity, in turn, can worsen PCOS symptoms, such as further metabolic problems and reproductive abnormalities [44]. BW alteration after EV protocol for PCOS induction in rats was controversial. A similar protocol as in our study, performed in adult virgin rats, resulted in BW decrease compared with the control group [45] by activation of the sympathetic nervous system and increasing the metabolic rate and fat consumption. However, in our study, BW was significantly increased in the EV group, while the combined protocol further increased BW compared with the CTRL as well as the EV group. It was previously shown that EV led to increase of BW [46], as we also registered in our study. Furthermore, higher calorie intake during the 60 days also increased BW in the EV + HFD group compared with CTRL, as expected. An additional effect of HFD and EV protocol was not investigated previously. It is known that obesity and PCOS share several comorbidities, while adiposity positively correlates with symptom severity [47]. Several mechanisms whereby weight gain and obesity contribute towards development of PCOS, and vice versa, were described [48]. Moreover, diet itself plays an important role in the pathogenesis of PCOS [49], while experimental rodent models of PCOS strongly confirmed that anovulation and cystic follicles formation associated with high androgen levels could be observed after diet manipulations such as HFD or HFHS protocols [20,42]. Insulin resistance, found in 50–70% of PCOS patients, itself influences androgen production by ovaries by co-gonadotrophic effects with LH on androgen synthesis in the ovaries, and releasing androgens from theca cells. Obesity and weight gain are linked to PCOS by the effects of weight gain on insulin resistance and hyperinsulinemia, as well as the dysmetabolic and steroidogenic effects of impaired PI3-kinase and intact MAP kinase post-receptor insulin pathways, respectively [48]. In this manner, effective weight loss in obese and overweight PCOS patients exerts the effects on metabolic health and reproductive function by increasing insulin sensitivity and blood insulin levels [48]. Some studies have shown that when carbohydrate intake is less than 45% of the total daily caloric intake, the diet may be effective in reducing body mass index as well as serum total cholesterol levels in individuals with PCOS [50]. Furthermore, studies show that maintaining a low-carb diet for more than a month can significantly increase levels of follicle-stimulating hormone and sex hormone-binding globulin [51]. Although some evidence points to an effect of a low-carbohydrate diet on PCOS, the definitive mechanisms explaining the relationship are still not fully elucidated. Metformin is well-known to have similar weight-reducing effects. Some studies have compared the effects of dietary modification with the effects of combined treatment involving metformin and dietary modification for PCOS. Dietary modification has been shown to reduce insulin resistance and increase serum sex hormone-binding globulin levels compared with metformin [52]. Although the EV model is thought to be inappropriate to study the hypothalamic–pituitary–ovarian axis, due to progressive degeneration of the hypothalamus and impaired pituitary response, ovarian anatomy and physiology resemble that of PCOS patients indeed [53]. Our study showed increased ovarian weight and ovarian index in both applied protocols compared with control values, while HFD did not influence EV-related changes regarding these parameters. Similar results were observed in previous studies investigating PCOS-related manifestations in rats [54,55], while other studies registered lower ovarian weight [23,45,56] after the EV protocol. The observed differences could be related to alteration of BW along with ovarian weight: our protocol resulted in BW and ovarian weight increase while the mentioned opposite results from other authors revealed BW decrease along with ovarian weight decrease. These controversies confirmed once again the pertinence of HFD in addition to EV protocol to produce similar manifestations as PCOS in women.

Estrus cycle impairments were one of the most prominent features in animal models of PCOS. Cycle cessation, as well as cycle impairment with different deviations from regular 4 to 5 days of duration, was observed in the experimental protocol after successful PCOS induction. Regarding the EV protocol, it was shown that estrus cycle was arrested in the estrus phase, with persistent vaginal cornification (PVC) observed at cytological microscopic analysis [57], or in persistent proestrus/estrus phase [45]. In the last 12 days of the protocol, we registered PVC in all animals as confirmed previously [46]. Interestingly, adding the HFD protocol to EV also resulted in persistent estrus phase in rats. When rats receive EV injections, the sympathetic nervous system is activated even before PCOS is induced and the cysts are formed [58,59]. The immune and endocrine systems are both severely compromised, while electroacupuncture treatments were shown to correct these issues by reducing sympathetic nervous system hyperactivity [39]. Both experimental and interventional research have revealed evidence of a causal connection between obesity and ovulatory failure. In murine studies, an obesogenic diet alone led to PCOS-like alterations, and obese mice showed greater abnormalities in their estrus cycles and higher levels of testosterone; obesity alters expression of ovarian inflammatory and steroidogenic pathway genes in ways which could adversely affect ovarian function [20,60]. Moreover, the Lee–Boot effect, which describes that in the absence of male rodents in the environment, female cycles are stopped or slowed down, could be neglected in our case; we concluded that cyclicity was ceased following the applied protocols (EV and HFD).

Hormone level alterations revealed that both testosterone and estradiol values were higher after both protocols were performed. These results are in accordance with the study by Mehraban et al. [46]. However, the EV + HFD protocol increased the level of estradiol compared with the EV group. The effects of higher calorie intake to increased estradiol levels were observed previously [61], while in our study, synergistic effects of the applied protocols were registered, which accentuate the significance of HFD addition to the EV protocol. The opposite situation was observed regarding progesterone levels: significant decrease after the EV and EV + HFD protocols, while between the applied protocols, we did not observe significant difference. Our results regarding the EV protocol to progesterone levels were similar to a previous investigation [46]. On the contrary, in rat and mice studies, after 120 days of the HFD protocol, progesterone level increased significantly [62,63]. The authors explained that increased progesterone levels may suppress LH release, although HFD mice were still cycling normally, suggesting the occurrence of ovulation. The particular role of progesterone signaling under HFD conditions will require further study. It is known that progesterone inhibits estradiol-induced LH surge during proestrus as well as GnRH/LH pulses via actin on kisspeptin neurons [64,65]. Further studies are needed to explore P inhibitory effects in the state of HFD protocols, while our results should be attributed to the EV administration protocol.

PCOS represents a heterogeneous disorder with a variety of reproductive and metabolic issues. Literature data showed numerous protocols for PCOS induction in rodents where scientists tried to resemble its complex and multifaceted appearance. Regarding the EV protocol applied alone, there has been a spectrum of different results observed, from those that suggest that administration of EV is not appropriate to establish metabolic profile with impaired glycoregulation similar to PCOS, according to standard biochemical techniques for glucose measurements [39,66], to those that showed elevated fasting blood glucose and impaired glycoregulation with insulin resistance [54,67]. In addition, Danesa et al. found elevated fasting blood glucose in the PCOS group, but without difference after OGTT between the PCOS and control group [68]. In our study, the EV protocol alone did not influence blood glucose levels in basal conditions (fasting blood glucose levels) nor in OGTT. We registered higher blood glucose levels after 30 min of OGTT in the EV + HFD group compared with control values. The EV + HFD group showed a higher level of blood glucose compared with the EV group after 30 and after 60 min of OGTT. After 120 min, blood glucose levels were similar in all investigated groups. Our results confirmed once again that HFD aggravates the metabolic profile in EV-induced PCOS in rats.

Ultrasonographical analysis confirmed a higher longitudinal diameter and ovarian volume in both applied protocols compared with the CTRL group, with no observed difference between those protocols. Notably, ovarian volume increased by 11.5% in the EV protocol, while this parameter was altered by 19.2% after the EV + HFD protocol. Microscopic analysis revealed a significantly higher number of cystic follicles and lower number of corpora lutea in both applied protocols compared with control values. However, addition of HFD to the EV protocol produced further increased number of cysts in the EV + HFD group compared with the EV group, while it did not influence the number of corpora lutea. Our results regarding the EV protocol are in line with previous reports [69,70]. The HFHS diet protocol produced similar changes in the number of cysts and corpora lutea, although ovary weights were lower [42]. Our results confirmed that the combination of two protocols led to increased ovarian volume, greater number of cystic follicles, and lower number of corpora lutea, as shown in PCOS women [71,72] and other PCOS animal models [73,74].

Mammalian reproduction relies on controlled oxidation, such as the formation of disulfide bonds in sperm nuclei and during ovulation process [75]. However, excessive oxidation results in oxidative stress, which compromises the reproductive system along with other functions in the organism. OS is one of the most investigated features that underlie PCOS pathophysiology in women [76] as well as in rodent experimental models [77]. Moreover, in the physiological conditions during ovulation and steroidogenesis in the ovary, ROS also rises. Detoxification of ROS is therefore crucial for oocyte maturation and fetal development. Due to the generation of MDA and lipid peroxidation, ROS disrupts ovarian function [11,78]. OS markers significantly altered between both applied protocols compared with control values and protocols for PCOS induction itself. Regarding the EV protocol, levels of O_2_^−^ significantly increased, which was further augmented by the HFD protocol. On the other hand, activity of SOD dropped as a result of neutralizing the higher O_2_^−^ levels. However, levels of H_2_O_2_ and CAT significantly decreased after the EV protocol, which could be explained by depletion of CAT in combating H_2_O_2_ levels [77], and these changes were further accentuated by adding HFD in the EV protocol, although activity of CAT did not differ between the EV and EV + HFD group. Index of lipid peroxidation was not altered by EV treatment, while the combination of EV and HFD produced significant increase of TBARS compared with the control and EV group. Such an increase of lipid peroxidation after the HFD protocol was expected, considering the previous reports regarding HFD [79]. Lipid peroxidation is responsible for gonadotropin receptors impairment, which reduces steroidogenesis in the corpus luteum [75]. Furthermore, levels of nitrites were higher, and alterations were in the same manner as TBARS. Similar results were also confirmed in the previous reports on male rats [80,81], as well as in PCOS rat models investigated in our previous studies [12,26,27]. However, our previous investigations regarding PCOS considered different methodology (androgens such as testosterone-enanthate and DHEA), while here for the first time, we evaluated the EV protocol combined with HFD. The results strongly confirmed involvement of OS in PCOS-related reproductive and metabolic changes.

Glutathione in its reduced form significantly increased after the EV protocol, while decreased in the EV + HFD compared with the EV group. Such a change could be explained by depletion of GSH, while all other prooxidative parameters increased in the EV + HFD group. As HFD is shown to reduce GSH levels in rat erythrocytes [82], we concluded that hormone-related changes, induced by EV alone were initiators of augmented synthesis of GSH, which was further depleted by adding HFD for 60 days.

## 5. Conclusions

Summarizing the obtained results, we can conclude that the EV protocol applied along with HFD represent a valuable novel experimental model to study reproductive and metabolic features in PCOS rat models. OS, promoted by EV as well as HFD, lies in many molecular pathways in PCOS-related alterations in the female gender. Using this model, the future researcher minimizes stressful daily animal handling and avoids parenteral or oral route of administration of various drugs to induce PCOS, while obtaining the fully developed PCOS characteristics, including estrus cycle impairment, cystic ovarian structure, enlarged ovarian volume, impaired hormonal and oxidative status, and glycoregulation.

## Figures and Tables

**Table 1:** Parameters of OS in rat’s plasma and erythrocyte lysates (n = 6).

Parameter	CTRL	EV	EV + HFD
TBARS	0.90 ± 0.00	0.91 ± 0.02	1.00 ± 0.02 **^,#^
NO_2_^−^	2091.63 ± 65.64	2235.33 ± 30.71 *	2418.95 ± 8.75 **^,##^
O_2_	2.14 ± 0.46	2.86 ± 0.22	4.61 ± 0.80 **^,#^
H_2_O_2_	8468.85 ± 459.89	4994.45 ± 398.99 **	4263.38 ± 54.26 **
SOD	25.78 ± 3.27	23.06 ± 3.27	12.21 ± 2.78 *^,#^
CAT	338.71 ± 14.22	223.50 ± 24.89 **	249.79 ± 25.81 *
GSH	65,706.28 ± 1412.98	84,207.19 ± 5253.47 *	62,903.11 ± 7800.83 ^#^

Values are presented as means ± SEM. * represents significant difference compared to CTRL at level *p* < 0.05, ** represents significant difference compared to CTRL at level *p* < 0.01, ^#^ represents significant difference compared to CTRL at level *p* < 0.05, ^##^ represents significant difference compared to CTRL at level *p* < 0.01.

## Data Availability

The data presented in this study are available on request from the corresponding author.
